# Risk factors for and prediction of mortality in critically ill medical–surgical patients receiving heparin thromboprophylaxis

**DOI:** 10.1186/s13613-016-0116-x

**Published:** 2016-02-27

**Authors:** Guowei Li, Lehana Thabane, Deborah J. Cook, Renato D. Lopes, John C. Marshall, Gordon Guyatt, Anne Holbrook, Noori Akhtar-Danesh, Robert A. Fowler, Neill K. J. Adhikari, Rob Taylor, Yaseen M. Arabi, Dean Chittock, Peter Dodek, Andreas P. Freitag, Stephen D. Walter, Diane Heels-Ansdell, Mitchell A. H. Levine

**Affiliations:** Department of Clinical Epidemiology and Biostatistics, McMaster University, Hamilton, ON Canada; Centre for Evaluation of Medicines, St. Joseph’s Healthcare Hamilton, McMaster University, 25 Main St. West, Suite 2000, 20th Floor, Hamilton, ON L8P 1H1 Canada; Department of Medicine, McMaster University, Hamilton, ON Canada; Duke Clinical Research Institute, Duke University, Durham, NC USA; Interdepartmental Division of Critical Care Medicine, University of Toronto, Toronto, ON Canada; Critical Care Medicine, St. Michael’s Hospital, Toronto, ON Canada; School of Nursing, McMaster University, Hamilton, ON Canada; Department of Critical Care Medicine, Sunnybrook Health Sciences Centre, Toronto, ON Canada; Mercy Clinic Adult Critical Care, Mercy Hospital Saint Louis, Saint Louis, MO USA; King Saud bin Abdulaziz University for Health Sciences and King Abdullah International Medical Research Center, Riyadh, Saudi Arabia; Critical Care Medicine, Vancouver Coastal Health Research Institute, Vancouver, BC Canada; Center for Health Evaluation and Outcome Sciences and Division of Critical Care Medicine, St. Paul’s Hospital and University of British Columbia, Vancouver, BC Canada

**Keywords:** Prediction model, Critical care, APACHE, Intensive care unit, Mortality

## Abstract

**Background:**

Previous studies have suggested that prediction models for mortality should be adjusted for additional risk factors beyond the Acute Physiology and Chronic Health Evaluation (APACHE) score. Our objective was to identify risk factors independent of APACHE II score and construct a prediction model to improve the predictive accuracy for hospital and intensive care unit (ICU) mortality.

**Methods:**

We used data from a multicenter randomized controlled trial (PROTECT, Prophylaxis for Thromboembolism in Critical Care Trial) to build a new prediction model for hospital and ICU mortality. Our primary outcome was all-cause 60-day hospital mortality, and the secondary outcome was all-cause 60-day ICU mortality.

**Results:**

We included 3746 critically ill non-trauma medical–surgical patients receiving heparin thromboprophylaxis (43.3 % females) in this study. The new model predicting 60-day hospital mortality incorporated APACHE II score (main effect: hazard ratio (HR) = 0.97 for per-point increase), body mass index (BMI) (main effect: HR = 0.92 for per-point increase), medical admission versus surgical (HR = 1.67), use of inotropes or vasopressors (HR = 1.34), acetylsalicylic acid or clopidogrel (HR = 1.27) and the interaction term between APACHE II score and BMI (HR = 1.002 for per-point increase). This model had a good fit to the data and was well calibrated and internally validated. However, the discriminative ability of the prediction model was unsatisfactory (*C* index < 0.65). Sensitivity analyses supported the robustness of these findings. Similar results were observed in the new prediction model for 60-day ICU mortality which included APACHE II score, BMI, medical admission and invasive mechanical ventilation.

**Conclusion:**

Compared with the APACHE II score alone, the new prediction model increases data collection, is more complex but does not substantially improve discriminative ability.

*Trial registration*: ClinicalTrials.gov Identifier: NCT00182143

## Background

Mortality rates in critically ill patients are substantial, ranging from 5 to 40 %, depending on case mix [1–3]. Predicting mortality in critically ill patients is challenging, but can be helpful for general counseling, triaging, treatment decisions and end of life discussions [4, 5].

The Acute Physiology and Chronic Health Evaluation (APACHE) prognostic scoring system is a well-established, validated tool for assessing the severity of illness and predicting hospital mortality using data obtained in the first 24 h of ICU admission [6–11]. To increase predictive accuracy, APACHE has developed four generations of models [12–15]. Nevertheless, there may be wide variation and limited validation in the ability of APACHE system to predict mortality in different countries and populations [16–20]. Siontis et al. reported a median AUC (the area under the receiver operating characteristic curve) of 0.77 for APACHE II model after conducting a systematic evaluation of predictive tools for all-cause mortality in critically ill patients [21]. Furthermore, the updated APACHE III and IV models include substantially more variables than APACHE II, with a correspondingly increased data collection burden [7, 22].

Advances in prognostic science have identified additional risk factors for mortality for critically ill patients that are independent of measures of illness severity, such as body mass index (BMI) [23, 24] and sex [25–27]. Moreover, some evidence suggests that prediction models for mortality should be adjusted for the use of vasopressors [28], prothrombin index [29, 30] and platelet count [31, 32]. Therefore, given the imperfect accuracy of the APACHE system and other potential risk factors for death, we aimed to identify risk factors independent of APACHE II score and construct and validate a new mortality prediction model that would combine the APACHE II score with these additional factors. The primary objective of this study was to improve the accuracy of a prediction model for 60-day hospital mortality in critically ill medical–surgical patients, based on the data from a multicenter randomized controlled trial, PROTECT (Prophylaxis for Thromboembolism in Critical Care Trial). Our secondary objective was to construct a prediction model for 60-day ICU mortality.

## Methods

In this study, we followed the TRIPOD (transparent reporting of a multivariable prediction model for individual prognosis or diagnosis) statement [33] to report the prediction model including model development, model performance and model validation.

### Patients and settings

PROTECT (ClinicalTrials.gov Identifier: NCT00182143) was an international randomized controlled trial (RCT) that was conducted in 67 ICUs in academic and community hospitals from 2006 to 2010 in Canada, Australia, Brazil, Saudi Arabia, the USA and the UK, as described elsewhere [34]. The trial compared the effect of unfractionated heparin (UFH) 5000 IU twice daily versus the low molecular weight heparin (LMWH) dalteparin 5000 IU once daily plus once-daily placebo on the primary outcome of proximal leg deep vein thrombosis.

Non-trauma medical–surgical critically ill patients were enrolled if they were at least 18 years of age, weighed ≥45 kg and were expected to remain in the ICU for at least 3 days. Exclusion criteria were: admission diagnoses of major trauma, neurosurgery or orthopedic surgery, uncontrolled hypertension (systolic blood pressure >180 mm Hg or diastolic blood pressure >110 mm Hg) for at least 12 h, major bleeding within the last week unless definitively treated, hemorrhagic stroke, coagulopathy (international normalized ratio >2 times upper limit of normal or activated partial thromboplastin time >2 times the upper limit of normal), severe thrombocytopenia (platelet count <75 × 10^9^/L), need for therapeutic anticoagulation, heparin administration in the ICU for at least 3 days, contraindication to heparin or blood products, pregnancy, life-support limitation, life expectancy ≤7 days or enrollment in another related trial [34, 35]. All patients, families, clinicians, research personnel and the trial biostatistician were blind to treatment allocation. Patients were followed up to death or hospital discharge.

### Outcome measures

During the trial follow-up, the vital status was documented in the ICU and in hospital. In this study, the primary outcome was 60-day hospital mortality. Patients survived longer than 60 days in hospital or discharged from hospital were censored. The secondary outcome was 60-day ICU mortality, and patients survived long than 60 days in ICU or discharged from the ICU were censored.

### Potential predictors

Based on the data recorded in PROTECT and our a priori plan, potential risk factors for death included baseline variables (APACHE II score, sex, BMI, history of malignancy, type of admission and diagnosis of sepsis on admission), use of UFH and interventions within the first 24 h of ICU admission (use of inotropes or vasopressors, invasive mechanical ventilation, dialysis and pharmacologic cointerventions). The APACHE II score [13] has three parts: an acute physiology score (up to 60 points), an age point (0–6) and a chronic health score (0–5). The acute physiology score is composed of 12 physiologic variables: creatinine (0–8 points); Glasgow Coma Scale (0–12 points); ten other variables including temperature, mean arterial pressure, heart rate, respiratory rate, oxygenation, arterial pH, serum sodium, potassium, hematocrit and white blood cell count (0–4 points each). The maximum total APACHE II score is 71 points, and a higher score indicates a higher predicted probability of death. The type of admission was categorized as either surgical or medical. Pharmacologic cointerventions included the use of a statin, and acetylsalicylic acid or clopidogrel.

### Statistical analyses

In this study, all analyses were conducted using STATA version 12 (Stata Corp., College Station, TX, USA). Data were summarized using the mean and standard deviation (SD), or median and interquartile range (IQR) or frequency and percentages. Comparisons between the patients who died and survived for the duration of hospital stay were made by using Student’s *t* test for continuous variables and Chi-square test for categorical variables, respectively. If <10 % of observations on a variable were missing, we imputed the missing values using the mean or median. If ≥10 % of data were missing, multiple imputations were performed, assuming they were missing at random [36].

### Identification of risk factors independent of APACHE II score and model development

To identify risk factors independent of APACHE II score, data were first randomly split into a training (derivation) set and a validation set stratifying by participating trial centers. The derivation set and validation set had an approximately equal sample size. In the derivation set, to avoid multicollinearity, we pruned the candidate predictors of those with a variance inflation factor (VIF) of no less than 4 [37, 38]. Cox proportional hazards regression was conducted to examine associations with death using the backward elimination approach [37], after adjustment for the APACHE II score, with a two-sided alpha value of 0.05. Hazard ratios (HRs) were used to quantify the relationship between risk factors and death. Both a statistical test of proportional hazards assumption and a graphical examination using Schoenfeld residuals were performed to test the proportional hazards assumption of the Cox regression models [39].

In the derivation set, the new prediction model for 60-day hospital mortality was constructed by combining the APACHE II score and the other risk factors identified above into a Cox regression model. Additionally, all the two-way interactions between the predictors in the new prediction model were tested. Significant interactions with an a priori alpha value of 0.05 were then added into the model to finalize the prediction model.

For 60-day ICU mortality, identification of risk factors independent of APACHE II score and construction of a new prediction model were performed in the whole dataset following the same process.

### Model performance

For succinctness, we defined three models for hospital and ICU mortality in this study: Model 1 which included the APACHE II score only; Model 2 that included the other risk factors only; and Model 3, as the new prediction model, which combined APACHE II score and the other risk factors. To assess the calibration of all the three models for 60-day hospital mortality in the derivation set, we calculated the standardized mortality ratio (SMR) by dividing the observed death risk by the predicted mortality. To obtain the 95 % confidence intervals (CIs) for SMRs, first we treated the observed mortality as a Poisson variable, and then divided its 95 % confidence limits by the predicted mortality [40]. For Model 1 and Model 3, we also compared and plotted the predicted and observed risks of death across each 10th of observed risk [41], in which the observed risk was obtained from the Kaplan–Meier product-limit estimate.

Model goodness-of-fit was evaluated using a Gronnesby and Borgan test with ten groups based on the predicted risk score, where a nonsignificant result indicated no evidence of lack of fit to the data [42]. The Akaike information criterion (AIC) was used to evaluate and compare the goodness-of-fit between the three models; a smaller AIC value indicated a better model [43]. The likelihood ratio test was also performed for model comparison. To measure discrimination, we calculated a Harrell’s *C* index for each model [37, 44].

For 60-day ICU mortality, performance of the three models was assessed and compared using the whole dataset.

### Model validation for hospital mortality

We used the validation set to assess the internal validation of all the three models [45]. The evaluation of calibration, goodness-of-fit and discrimination was again performed in the validation set [45, 46].

### Sensitivity analyses

To assess the robustness of findings, we performed a sensitivity analysis by using restricted cubic splines for continuous predictors in the new model [37]. Another sensitivity analysis was conducted using data for 30-day hospital and 30-day ICU mortality, and 90-day hospital and 90-day ICU mortality.

### Exploratory analysis for hospital mortality

We applied and compared Model 3 and Model 1 in different countries for 60-day hospital mortality in the whole dataset, as an exploratory analysis. Model performance was assessed separately in Canada, Saudi Arabia and Brazil, USA and UK, and Australia.

## Results

### Baseline characteristics of participants

There were 3746 patients included for analyses. The mean age at baseline was 61.4 (SD: 16.5) years, and 43.3 % were females. The median survival of the 588 (15.7 %) patients who died in the ICU was 10 days. The median survival of the 873 (23.3 %) patients who died during the hospital stay was 14 days.

**Fig. 1:**
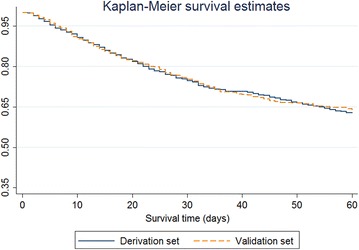
Kaplan–Meier survival curves for 60-day hospital mortality in derivation and validation sets

The data were randomly split into a derivation set (*n* = 1891) and a validation set (*n* = 1855). Figure 1 shows Kaplan–Meier survival curves of 60-day hospital mortality in the derivation and validation sets, with no evidence of significant difference between the two sets (*p* value = 0.94 for log-rank test). Table 1 compares the baseline characteristics between the survivors and non-survivors in the derivation and validation sets. In the derivation set, 22.6 % of participants (*n* = 428) died during the whole follow-up period and their median survival time was 14 days (IQR 7.5–28). The median follow-up for survivors (*n* = 1463) was 18 days (IQR 11–33). Non-survivors were significantly older than survivors (67.7 vs. 59.3 years). The survivors had significantly lower APACHE II scores but higher BMI than non-survivors (*p* value <0.001). There were more patients receiving UFH in non-survivors (54.7 %) than in survivors (49.1 %). More non-surviving patients were admitted to ICU with the diagnoses of sepsis and medical reasons (*p* value <0.001). Non-survivors were significantly more likely to receive inotropes or vasopressors, invasive mechanical ventilation, dialysis and acetylsalicylic acid or clopidogrel within the first 24 h of ICU admission (*p* value <0.05). Similar comparisons were also found in the validation set between non-survivors and survivors, except for the proportions of patients receiving UFH, invasive mechanical ventilation and dialysis, and the percentages of patients with malignancy and medical admission (Table 1).

**Table 1:** Baseline characteristics of survivors and non-survivors in hospital in derivation and validation datasets

Characteristics	Derivation set (*n* = 1891)			Validation set (*n* = 1855)		
	Survivors^a^ (*n* = 1463)	Non-survivors^b^ (*n* = 428)	*p* value	Survivors^c^ (*n* = 1410)	Non-survivors^d^ (*n* = 445)	*p* value
Age (year): mean (SD)	59.3 (16.92)	67.7 (14.58)	<0.001^e^	59.6 (16.07)	68.3 (14.40)	<0.001^e^
Gender: *n* (%)						
Male	830 (56.93)	231 (54.10)	0.301^f^	786 (56.22)	266 (59.91)	0.172^f^
Female	628 (43.07)	196 (45.90)		612 (43.78)	178 (40.09)	
BMI (kg/m^2^): mean (SD)	28.5 (7.69)	27.1 (6.93)	<0.001^e^	28.9 (8.39)	27.1 (6.78)	<0.001^e^
Use of thromboprophylaxis: *n* (%)						
Unfractionated heparin	718 (49.08)	234 (54.67)	0.042^f^	696 (49.36)	225 (50.56)	0.659^f^
Dalteparin	745 (50.92)	194 (45.33)		714 (50.64)	220 (49.44)	
APACHE II score: mean (SD)	20.8 (7.61)	24.1 (7.59)	<0.001^e^	20.6 (7.59)	24.5 (7.84)	<0.001^e^
History of malignancy: *n* (%)	50 (3.43)	21 (4.92)	0.155^f^	47 (3.36)	32 (7.21)	<0.001^f^
Medical admission: *n* (%)	1086 (74.23)	353 (82.48)	<0.001^f^	1046 (74.18)	346 (77.75)	0.129^f^
Diagnosis of sepsis on admission: *n* (%)	208 (14.27)	90 (21.08)	<0.001^f^	177 (12.66)	74 (16.67)	0.032^f^
Intervention within the first 24 h on admission: *n* (%)						
Inotropes or vasopressors	614 (42.20)	241 (56.44)	<0.001^f^	579 (41.42)	243 (54.73)	<0.001^f^
Invasive mechanical ventilation	1207 (82.96)	373 (87.35)	0.030^f^	1149 (82.19)	380 (85.59)	0.097^f^
Dialysis	57 (3.92)	45 (10.54)	<0.001^f^	87 (6.22)	39 (8.78)	0.063^f^
Acetylsalicylic acid or clopidogrel	290 (19.93)	113 (26.46)	0.004^f^	289 (20.49)	112 (25.23)	0.029^f^
Statin	169 (11.62)	62 (14.52)	0.108^f^	199 (14.23)	59 (13.29)	0.617^f^

^a^Median follow-up: 18 days; interquartile range (IQR): 11–33 days

^b^Median survival time: 14 days; IQR 7.5–28 days

^c^Median follow-up: 19 days; IQR 11–37 days

^d^Median survival time: 15 days; IQR 7–29 days

^e^Student’s *t* test

^f^Chi-square test

### Model construction

Table 2 shows the predictors and their HRs included in the new model (Model 3) for 60-day hospital and 60-day ICU mortality. Based on the derivation set, BMI, medical admission, use of inotropes or vasopressors and acetylsalicylic acid or clopidogrel were significant risk factors for 60-day hospital mortality independently of APACHE II score; all of them except BMI increased the risk of hospital death. Model 3 for hospital mortality included APACHE II score (main effect: HR = 0.97, 95 % CI 0.92–1.02 for per-point increase), BMI (main effect: HR = 0.92, 95 % CI 0.88–0.97 for per-point increase), medical admission (HR = 1.67, 95 % CI 1.29–2.17), use of inotropes or vasopressors (HR = 1.34, 95 % CI 1.10–1.65), acetylsalicylic acid or clopidogrel (HR = 1.27, 95 % CI 1.02–1.59) and the interaction term between APACHE II score and BMI (HR = 1.002, 95 % CI 1.000–1.004 for per-point increase) (Table 2).

**Table 2:** Predictors for 60-day hospital mortality in the derivation dataset and for 60-day ICU mortality in the whole dataset

Predictors	Hospital mortality (*n* = 1891)^a^		ICU mortality (*n* = 3746)^b^	
	HR (95 % CI)	*p* value	HR (95 % CI)	*p* value
BMI	0.92 (0.88–0.97)	0.003	0.98 (0.96–0.99)	<0.001
Medical admission	1.67 (1.29–2.17)	<0.001	1.39 (1.11–1.72)	0.003
Inotropes or vasopressors	1.34 (1.10–1.65)	0.005	–^c^	–^c^
Acetylsalicylic acid or clopidogrel	1.27 (1.02–1.59)	0.035	–^c^	–^c^
APACHE II score	0.97 (0.92–1.02)	0.241	1.04 (1.03–1.05)	<0.001
APACHE II score*BMI	1.002 (1.000–1.004)	0.038	–^c^	–^c^
Invasive mechanical ventilation	–^d^	–^d^	0.75 (0.58–0.97)	0.027

^a^There were 390 60-day deaths in hospital in derivation cohort

^b^There were 573 60-day deaths in ICU in the whole cohort

^c^Not included in the model for ICU mortality; no interaction term in the model for ICU mortality

^d^Not included in the model for hospital mortality

Significant risk factors for 60-day ICU mortality independent of APACHE II score were BMI, medical admission and invasive mechanical ventilation. Model 3 for ICU mortality included APACHE II score, BMI, medical admission and invasive mechanical ventilation, with a HR of 1.04, 0.98, 1.39 and 0.75, respectively. No significant interaction terms were identified for Model 3 (Table 2).

### Model performance

**Fig. 2:**
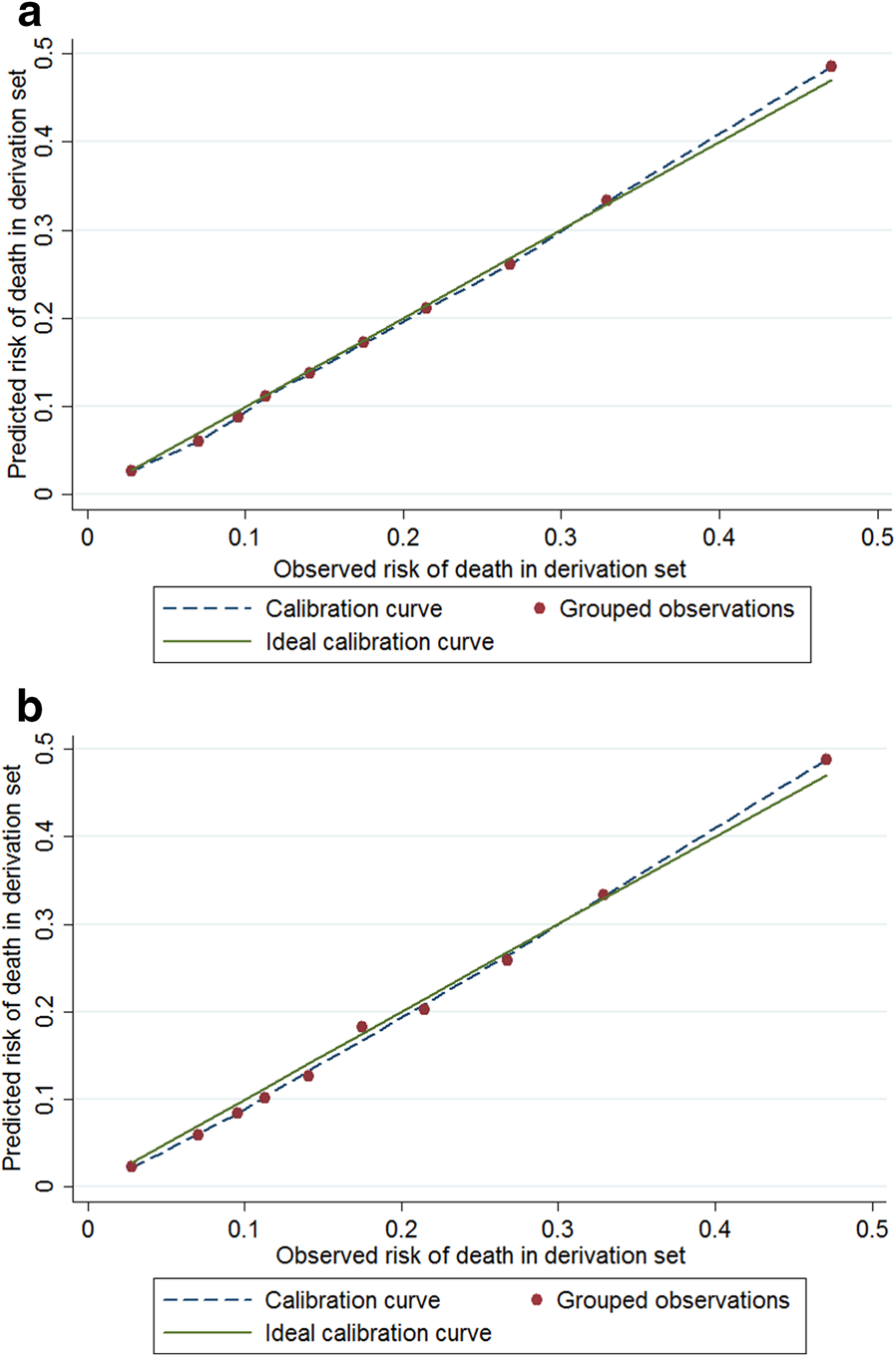
Observed versus expected in *derivation set* for 60-day *hospital* mortality: **a** results from Model 1; **b** results from Model 3 (*solid diagonal line* represents ideal calibration)

Results and comparison of the three models for 60-day hospital mortality are shown in Table 3. In the derivation set, the goodness-of-fit test indicated no evidence of lack of fit to the data for Model 1 (*p* value = 0.68) for hospital mortality. However, the discriminative ability of Model 1 was poor (*C* index = 0.58). No evidence for the inaccurate overall prediction of mortality by Model 1 was found, given that the SMR was not significantly different from 1 (SMR = 1.003, 95 % CI 0.959–1.050) (Table 3). Figure 2a displays predicted and observed hospital mortality in the derivation set across each 10th of the observed risk of death for Model 1, indicating Model 1 was well calibrated. Similarly, Model 2 was a good fit and well calibrated in the derivation set, but its discriminative power was not high (*C* index = 0.62). Model 3 had a *C* index of 0.64 and a SMR of 1.006 (95 % CI 0.961–1.052) (Table 3). The difference in *C* indices between Model 3 and Model 1 was significant (*p* value <0.001). Figure 2b shows predicted versus observed hospital mortality in the derivation set, which justified the calibration of Model 3. The smallest AIC was observed in Model 3, indicating that Model 3 performed better than the other two models (Table 3). Likelihood ratio test also implied that Model 3 was a better fit than Model 1 and Model 2 (*p* values <0.001).

**Table 3:** Comparing three models in model performance for 60-day hospital mortality in the derivation and validation dataset and for 60-day ICU mortality in the whole dataset

Model performance	Goodness-of-fit		*C* index	SMR (95 % CI)
	*p* value^a^	AIC		
Hospital mortality—derivation set (*n* = 1891)				
Model 1 (including APACHE II scores only)	0.68	5704	0.58	1.003 (0.959–1.050)
Model 2 (including the other risk factors only)^b^	0.16	5359	0.62	1.002 (0.956–1.049)
Model 3 (including both the other risk factors and APACHE II scores)^c^	0.90	5329	0.64	1.006 (0.961–1.052)
Hospital mortality—validation set (*n* = 1855)				
Model 1 (including APACHE II scores only)	0.37	5912	0.60	0.933 (0.890–0.978)
Model 2 (including the other risk factors only)^b^	0.80	5765	0.59	1.019 (0.972–1.067)
Model 3 (including both the other risk factors and APACHE II scores)^c^	0.88	5567	0.64	1.011 (0.966–1.060)
ICU mortality—the whole set (*n* = 3746)				
Model 1 (including APACHE II scores only)	0.75	8180	0.61	1.003 (0.972–1.036)
Model 2 (including the other risk factors only)^d^	0.24	7821	0.58	1.001 (0.969–1.034)
Model 3 (including both the other risk factors and APACHE II scores)^e^	0.74	7778	0.64	1.004 (0.972–1.038)

*AIC* Akaike information criterion, *SMR* standardized mortality ratio

^a^Based on Groennesby and Borgan test

^b^The other risk factors included BMI, medical admission, inotropes or vasopressors and acetylsalicylic acid or clopidogrel

^c^Model 3 consisted of BMI, medical admission, inotropes or vasopressors, acetylsalicylic acid or clopidogrel, APACHE II score and the interaction between BMI and APACHE II score

^d^The other risk factors included BMI, medical admission and invasive mechanical ventilation

^e^Model 3 consisted of BMI, medical admission, invasive mechanical ventilation and APACHE II score

**Fig. 3:**
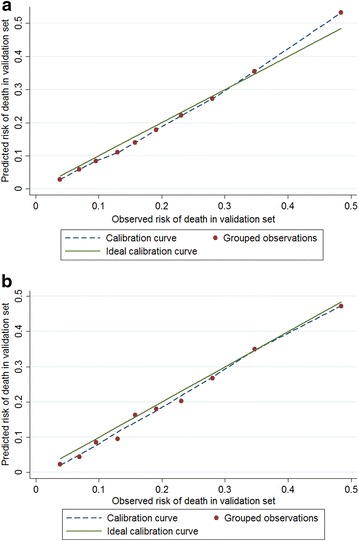
Observed versus expected in *validation set* for 60-day *hospital* mortality: **a** results from Model 1; **b** results from Model 3 (*solid diagonal line* represents ideal calibration)

When the models were applied to the validation set, findings were unchanged. All the three models were well calibrated (Table 3; Fig. 3a for Model 1 and Fig. 3b for Model 3, respectively); nevertheless, their discriminative power was not satisfactory (Table 3). Results from AIC and likelihood ratio tests presented that Model 3 was better than Model 1 and Model 2.

**Fig. 4:**
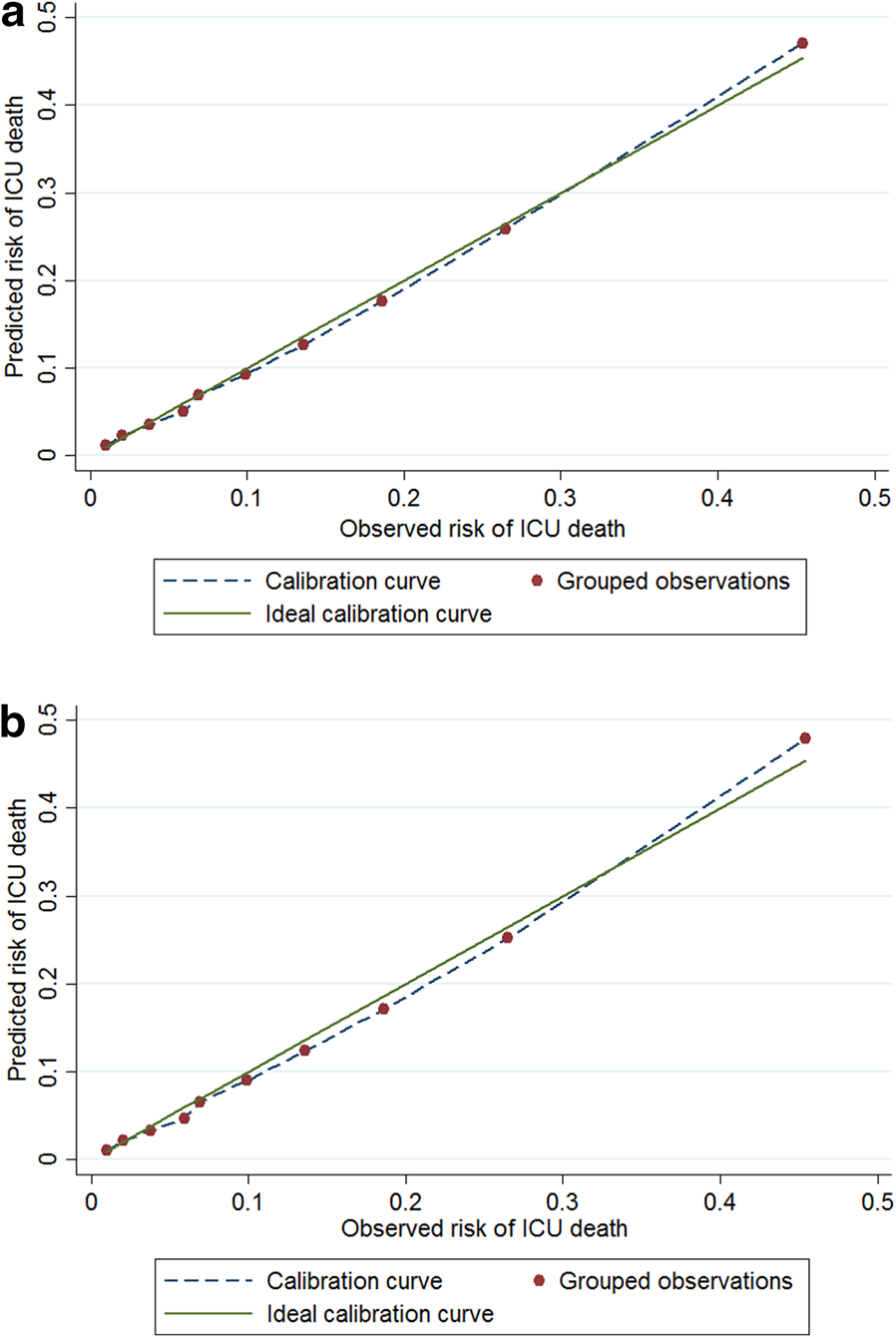
Observed versus expected in the *whole dataset* for 60-day *ICU* mortality: **a** results from Model 1; **b** results from Model 3 (*solid diagonal line* represents ideal calibration)

Table 3 also displays results for 60-day ICU mortality using the whole dataset. The SMR was 1.003 and 1.004 for Model 1 and Model 3, respectively. Figure 4a and b also support the calibration of Model 1 and Model 3, respectively. The *C* index was not high, with a discriminative value of 0.61 and 0.64 for Model 1 and Model 3, respectively. No significant difference in *C* indices between Model 3 and Model 1 was observed (*p* value = 0.16).

Results from sensitivity analysis using restricted cubic splines for BMI, APACHE II score and the interaction between them displayed similar findings from Model 3 for 60-day hospital mortality, where the interior knots were located on 25 and 30 for BMI, and the medians for APACHE II score (21) and the interaction term (569), respectively (Table 4). Findings were also in good agreement with Model 3 for 60-day ICU mortality when restricted cubic splines were used for BMI and APACHE II score (Table 4). Similar results of model construction and model performance were observed in another sensitivity analysis limiting data to 30-day hospital and 30-day ICU mortality (Appendix Tables 6, 7) and restricting data to 90-day hospital and 90-day ICU mortality (Appendix Tables 8, 9).

**Table 4:** Sensitivity analyses of model performance in Model 3 using restricted cubic splines for continuous predictors^a^ for 60-day hospital and 60-day ICU mortality

Model performance	Goodness-of-fit		*C* index	SMR (95 % CI)
	*p* value^b^	AIC		
Hospital mortality in derivation set (*n* = 1891)^c^	0.54	5691	0.63	1.007 (0.963–1.054)
Hospital mortality in validation set (*n* = 1855)^c^	0.51	5924	0.62	0.988 (0.943–1.034)
ICU mortality in the whole set (*n* = 3746)^d^	0.69	8177	0.64	1.005 (0.973–1.037)

*AIC* Akaike information criterion, *SMR* standardized mortality ratio

^a^Continuous predictors included BMI, APACHE score and the interaction between them for hospital mortality, and continuous predictors only included BMI and APACHE score for ICU mortality

^b^Based on Groennesby and Borgan test

^c^Model 3 for hospital mortality included BMI, medical admission, inotropes or vasopressors, acetylsalicylic acid or clopidogrel, APACHE II score and the interaction between BMI and APACHE II score

^d^Model 3 for ICU mortality consisted of BMI, medical admission, invasive mechanical ventilation and APACHE II score

### Exploratory analysis

An exploratory analysis was conducted by country for hospital mortality using the whole dataset (Table 5). Similar model performance was observed in different countries using Model 1 and Model 3. However, evidence indicated that Model 1 may under-predict risk of 60-day hospital death for patients in Saudi Arabia and Brazil (SMR = 1.155, 95 % CI 1.054–1.263).

**Table 5:** Exploratory analyses for model performance of Models 1 and 3 in different countries for 60-day hospital mortality using the whole dataset

Model performance	Goodness-of-fit		*C* index	SMR (95 % CI)
	*p* value^a^	AIC		
Model 1^b^				
Canada (*n* = 2456)	0.41	7639	0.61	0.981 (0.942–1.021)
Australia (*n* = 768)	0.11	1418	0.62	0.965 (0.897–1.037)
USA and UK (*n* = 109)	0.64	130	0.68	0.982 (0.804–1.186)
Saudi Arabia and Brazil (*n* = 413)	0.77	1713	0.55	1.155 (1.054–1.263)
Model 3^c^				
Canada (*n* = 2456)	0.88	7489	0.62	0.996 (0.956–1.037)
Australia (*n* = 768)	0.80	1349	0.63	0.968 (0.898–1.043)
USA and UK (*n* = 109)	0.41	125	0.71	0.981 (0.801–1.190)
Saudi Arabia and Brazil (*n* = 413)	0.25	1660	0.63	1.099 (0.943–1.311)

*AIC* Akaike information criterion, *SMR* standardized mortality ratio

^a^Based on Groennesby and Borgan test

^b^Model 1 included APACHE II score only

^c^Model 3 consisted of BMI, medical admission, inotropes or vasopressors, acetylsalicylic acid or clopidogrel, APACHE II score and the interaction between BMI and APACHE II score

## Discussion

### Main findings

Based on the data from an international thromboprophylaxis trial, we identified risk factors other than APACHE II score which predicted 60-day hospital mortality and 60-day ICU mortality. We constructed a new prediction model for mortality in critically ill patients receiving thromboprophylaxis. The new model was a good fit, well calibrated and internally validated. Results from sensitivity analyses supported the robustness of findings. However, the discriminative ability of the prediction model was not satisfactory.

In this study, we identified that higher BMI was significantly related to decreased risk of hospital and ICU mortality (Table 2), which was congruent with previous studies [47–49]. The potentially protective effect of increased BMI on survival has been termed the obesity paradox or reverse epidemiology [50], but whether the observed association is causative remains unresolved [49, 51, 52]. It has been postulated that higher body weight affords nutritional reserves that increase the chance of survival when patients are critically ill [53].

Medical admission was also found to be a significant independent risk factor for hospital and ICU death. Patients admitted for medical reasons may have more serious chronic morbidities not fully accounted for by APACHE chronic conditions, or have poorer prognoses when admitted to the ICU compared to those patients selected for surgery. Also, we found that some interventions within the first 24 h of ICU admission such as use of inotropes or vasopressors, and acetylsalicylic acid or clopidogrel, were associated with increased risk of hospital mortality, reflecting more severe illness. However, invasive mechanical ventilation within the first 24 h on admission was associated with 25 % decreased risk of ICU death (Table 2). Evidence suggests that not using invasive mechanical ventilation could have negative effects on outcome by postponing necessary intubation; therefore, early initiation of invasive mechanical ventilation may be related to decreased risk of death [54, 55].

### Implications of the study

Given the previously acknowledged limited predictive accuracy of the APACHE II system for mortality, we sought to build a new prediction model for mortality for critically ill medical–surgical patients. In this study, the model including APACHE II score only (Model 1) had surprisingly low discriminative ability (Tables 3, 5), which has been documented previously [21]. The model that combined additional baseline characteristics and early ICU interventions may better assess patients’ illness severity and thus improve the estimated risk of mortality, compared with the APACHE II score alone. Nevertheless, though the prediction model had a significantly higher *C* index than APACHE II score in predicting risk of hospital mortality, adding more information such as BMI, medical admission and early pharmacologic interventions increased the discriminative accuracy to only a small extent (Table 3). The simplicity of the APACHE II score is a major reason why it remains the most commonly used severity scoring system globally in clinical practice as well as health research [4]. Compared with the APACHE II score alone, the utilization of a new model which increases data collection, is more complex but does not substantially improve discriminative ability. Therefore, the use of the new model would be limited to situations where a clinician or health services investigator was sufficiently dissatisfied with the APACHE II and was requiring an even minimally better model to predict risk of death in critically ill patients.

### Comparison with other studies

Prediction models based on multivariate analyses typically use logistic regression analysis, due to the advantage of its simpler interpretation of the relationship between predictive factors and outcomes [56]. One study built a prediction model combining APACHE II score, a Model for End-Stage Liver Disease score, mechanical ventilation and sex using logistic regression in ICU patients with end-stage liver disease [57]. They found that the new model was more accurate than APACHE II score alone (the area under the receiver operating characteristic curves (AUC): 0.86 versus 0.76) in prediction of hospital mortality [57]. Another cohort study employed an assessment tool based on the PIRO (predisposition, insult, response and organ dysfunction) concept including comorbidities, old age, multilobar opacities in chest radiograph, shock, hypoxemia, bacteremia, acute renal failure and acute respiratory distress syndrome, to compare its model performance with APACHE II score in patients with community-acquired pneumonia [58]. The AUC of the PIRO score (0.88) was significantly higher than that of APACHE II score (0.75) in predicting 28-day ICU mortality [58]. Though it was difficult to directly compare our results with these models, given their different populations, settings, data and methodologies, these studies agreed with our findings in that adding more information to build a new model would likely outperform the APACHE II score alone.

### Limitations and strengths

This study was based on the data from a randomized thromboprophylaxis trial with strict inclusion and exclusion criteria, which therefore limits the generalizability of its findings. For instance, the mortality rate in this study may be lower than in other studies, because patients with poor life expectancy were excluded in the trial protocol [34, 35]. As well, the population upon which the new model was developed excluded patients who were at high risk of bleeding or if they were admitted to ICU because of major trauma, neurosurgery or orthopedic surgery [34, 35]. The latter criteria could also explain the apparent lower mortality associated with surgical patients compared to medical patients, as some of the more seriously ill surgical patients (e.g., patients with trauma, neurosurgical or orthopedic surgery) were excluded from the study. In addition, we could only use the data included in the original trial database, and we could not subsequently capture other potentially important indicators of illness severity including those that might have helped with the discrimination of this new model.

Strengths of this study include the international multicenter design, large sample size and standardized data collection. Moreover, we performed rigorous statistical analyses to build a new model and evaluate its performance. Evidence from internal validation and sensitivity analyses indicated that the findings were internally validated and robust. Similar results from explanatory analyses in different countries also suggested the generalizability and robustness of the model in this dataset using a heterogeneous group of patients.

## Conclusion

Using data from critically ill medical–surgical patients receiving heparin thromboprophylaxis, we identify additional risk factors for mortality independent of APACHE II score and construct a new model to predict risk of death. The new model combining APACHE II score and other risk factors is a good fit, well calibrated, but with unsatisfactory discriminative power. Compared with the APACHE II score alone, the new prediction model which increases data collection, is more complex but does not substantially improve discriminative ability.

## Appendix

See Tables 6, 7, 8 and 9.

**Table 6:** Predictors for 30-day hospital mortality in the derivation dataset and for 30-day ICU mortality in the whole dataset

Predictors	Hospital mortality (*n* = 1891)^a^		ICU mortality (*n* = 3746)^b^	
	HR (95 % CI)	*p* value	HR (95 % CI)	*p* value
BMI	0.97 (0.95–0.99)	<0.001	0.97 (0.96–0.99)	<0.001
Medical admission	1.86 (1.38–2.51)	<0.001	1.41 (1.12–1.78)	0.003
Inotropes or vasopressors	1.45 (1.16–1.82)	0.001	–^c^	–^c^
Acetylsalicylic acid or clopidogrel	1.22 (1.02–1.46)	0.037	–^c^	–^c^
APACHE II score	1.03 (1.01–1.04)	<0.001	1.04 (1.03–1.05)	<0.001
Invasive mechanical ventilation	–^d^	–^d^	0.77 (0.59–0.99)	0.042

^a^There were 332 deaths for 30-day hospital mortality in derivation cohort

^b^There were 522 deaths for 30-day ICU mortality in the whole cohort

^c^Not included in the model for ICU mortality

^d^Not included in the model for hospital mortality

**Table 7:** Results for sensitivity analyses of model performance in the new model for 30-day hospital mortality and 30-day ICU mortality

Model performance	Goodness-of-fit		*C* index	SMR (95 % CI)
	*p* value^a^	AIC		
Hospital mortality in derivation set (*n* = 1891)^b^	0.74	4682	0.63	1.005 (0.960–1.051)
Hospital mortality in validation set (*n* = 1855)^b^	0.76	4789	0.65	1.006 (0.961–1.053)
ICU mortality in the whole set (*n* = 3746)^c^	0.65	7559	0.64	1.003 (0.971–1.036)

*AIC* Akaike information criterion, *SMR* standardized mortality ratio

^a^Based on Groennesby and Borgan test

^b^The new model for 30-day hospital mortality included BMI, medical admission, inotropes or vasopressors, acetylsalicylic acid or clopidogrel and APACHE II score

^c^The new model for 30-day ICU mortality consisted of BMI, medical admission, invasive mechanical ventilation and APACHE II score

**Table 8:** Predictors for 90-day hospital mortality in the derivation dataset and for 90-day ICU mortality in the whole dataset

Predictors	Hospital mortality (*n* = 1891)^a^		ICU mortality (*n* = 3746)^b^	
	HR (95 % CI)	*p* value	HR (95 % CI)	*p* value
BMI	0.93 (0.88–0.97)	0.003	0.98 (0.96–0.99)	<0.001
Medical admission	1.67 (1.29–2.17)	<0.001	1.39 (1.12–1.73)	0.003
Inotropes or vasopressors	1.36 (1.11–1.67)	0.003	–^c^	–^c^
Acetylsalicylic acid or clopidogrel	1.27 (1.02–1.59)	0.035	–^c^	–^c^
APACHE II score	0.97 (0.92–1.02)	0.241	1.04 (1.02–1.05)	<0.001
APACHE II score*BMI	1.002 (1.000–1.004)	0.038	–^c^	–^c^
Invasive mechanical ventilation	–^d^	–^d^	0.75 (0.58–0.97)	0.026

^a^There were 405 deaths for 90-day hospital mortality in derivation cohort

^b^There were 581 deaths for 90-day ICU mortality in the whole cohort

^c^Not included in the model for ICU mortality

^d^Not included in the model for hospital mortality

**Table 9:** Results for sensitivity analyses of model performance in the new model for 90-day hospital mortality and 90-day ICU mortality

Model performance	Goodness-of-fit		*C* index	SMR (95 % CI)
	*p* value^a^	AIC		
Hospital mortality in derivation set (*n* = 1891)^b^	0.40	5521	0.63	1.006 (0.962–1.053)
Hospital mortality in validation set (*n* = 1855)^b^	0.62	5787	0.64	1.008 (0.963–1.055)
ICU mortality in the whole set (*n* = 3746)^c^	0.78	8144	0.63	1.004 (0.972–1.037)

*AIC* Akaike information criterion, *SMR* standardized mortality ratio

^a^Based on Groennesby and Borgan test

^b^The new model for 90-day hospital mortality included BMI, medical admission, inotropes or vasopressors, acetylsalicylic acid or clopidogrel, APACHE II score and the interaction between BMI and APACHE II score

^c^The new model for 90-day ICU mortality consisted of BMI, medical admission, invasive mechanical ventilation and APACHE II score

## Abbreviations

AIC: Akaike information criterion
APACHE: Acute Physiology and Chronic Health Evaluation
AUC: area under the receiver operating characteristic curves
BMI: body mass index
CI: confidence interval
HR: hazard ratio
IQR: interquartile range
ICU: intensive care unit
LMWH: low molecular weight heparin
PIRO: predisposition, insult, response and organ dysfunction
PROTECT: Prophylaxis for Thromboembolism in Critical Care Trial
RCT: randomized controlled trial
SMR: standardized mortality ratio
SD: standard deviation
TRIPOD: transparent reporting of a multivariable prediction model for individual prognosis or diagnosis
UFH: unfractionated heparin
VIF: variance inflation factor
